# Determinants of inappropriate complementary feeding among children 6–23 months of age in Dessie City Northeast Ethiopia: a case-control study

**DOI:** 10.1186/s40795-023-00779-w

**Published:** 2023-11-03

**Authors:** Meron Tadesse, Yeshimebet Ali Dawed, Zinabu Fentaw, Abel Endawike, Kidist Adamu

**Affiliations:** 1 Department of Nutrition, School of Public Health, College of Medicine and Health Sciences, Wollo University, Dessie, 1145 Ethiopia; 2 Department of Epidemiology and Biostatistics, School of Public Health, College of Medicine and Health Sciences, Wollo University, Dessie, Ethiopia; 3 Department of Health System Management, School of Public Health, College of Medicine and Health Sciences, Wollo University, Dessie, Ethiopia

**Keywords:** Inappropriate complementary feeding, Determinants, Case-control, Ethiopia

## Abstract

**Background:**

Inappropriate complementary feeding is one of the leading causes of malnutrition among children 6–23 months old and delays children’s growth milestone. The determinants of inappropriate complementary feeding practice have diverse natures, so that many of the previous studies fail to generate adequate evidence on it. This study aim to address the determinants of in appropriate complementary feedings at community level.

**Methods:**

A community-based unmatched case-control study design was carried out among children 6–23 months of age in Dessie City from April 13, 2021 to May 13, 2021. Nine kebeles were selected by simple random sampling method. One month prior to the data collection time survey was conducted and 482 samples were taken from the preliminary survey data; 241 cases and 241 controls by computer generated random numbers. Interviewer administered questionnaire was used to investigate potential determinants of inappropriate complementary feeding practice. Binary logistics regression was used to identify independent determinants.

**Results:**

Part working situation of mother [AOR = 0.21 CI: 0.08, 0.52] was negatively associated with inappropriate complementary feeding. Having no post-natal care visit [AOR = 4.062 CI:2.35,7.02], poor wealth status [AOR = 2.7 CI:1.09,6.68], food in-security [AOR = 4.49 CI:1.94,10.37], home delivery [AOR = 4.33 CI:1.43,13.15], having poor knowledge on infant and young child feeding [AOR = 5.94 CI:2.8,12.6], having no health education on complementary feeding [AOR = 2.54 CI:1.28,5.06] and father’s job [AOR = 2.2 CI:1.17,4.1] were found to be positively associated with inappropriate complementary feeding.

**Conclusion:**

Mothers’ work situation, fathers’ job, wealth index, food security, mothers’ knowledge on infant feeding, post-natal care, health education on infant feeding and place of delivery were independent determinants for inappropriate complementary feeding. Thus, interventions shall effectively address those factors to alleviate the problem.

## Introduction

Complementary feeding is defined as the process of starting solid, semisolid or soft food when breast milk alone is no longer sufficient to meet the nutritional requirements of infants and therefore other foods and liquids are needed, along with breast milk [1]. According to world health organization(WHO), inappropriate complementary feeding is when a child at the age of 6 to 23 months experience too early or too late initiation in complementary feeding, consumes less than the recommended number of diversified food groups which is less than five food groups, and consumes less than the recommended number of meal frequency based on their age. If either of them is not fulfilled it is termed as inappropriate complementary feeding [1, 2]. Only one in six children worldwide and one in ten in Eastern and South Africa are eating a minimum acceptable diet [3]. Only 21% of breastfed children in Nigeria consume the bare minimum of a supplementary feeding diet [4]. Just 7% of children in Ethiopia between the ages of 6 and 23 meet the minimum standards [5].

If children are not having the appropriate complementary feeding they will bear the brunt of chronic malnutrition and suffer the greatest consequences, that is, the highest risks of morbidity and mortality [6–8]. As it is a critical period of growth for the children they will end up in growth failure, micronutrient deficiencies, and emergence of common childhood diseases like diarrhea and respiratory tract infections [9]. Apart from contributing to childhood disease burden [10], early under nutrition has long lasting effects on physical as well as cognitive growth into adulthood [11].

Researches done in many areas show the strong association of complementary feeding with socio-demographic, household, obstetric, health service utilization and maternal knowledge related factors [12–17]. A recent systematic review conducted in Ethiopia suggested that maternal employment, improved knowledge on complementary feeding, frequent antenatal care (ANC) or postnatal care visits, and giving birth at the health facility were associated with the timely introduction of complementary foods [18].

Considering the consequences of inappropriate feeding and malnutrition there are internationally developed goals and declarations. These include sustainable development goals, scaling up nutrition and global nutrition monitoring frame work. Among the 17 goals in sustainable development goals 12 of them are related with nutrition [19]. In Ethiopia, several efforts have been carried out to alleviate the nutritional status of vulnerable groups which includes being member of internationally developed goals, preparing policies, strategies, conventions and national plans of actions. Among them national nutrition strategy I and II; seqota declarations are one of them [20, 21]. Despite these efforts several parts of the developing countries including Ethiopia complementary feeding continues as a challenge to good nutrition in children of 6–23 months [22].

Most of the researches done in Ethiopia on complementary feeding are carried out at district level [17, 23, 24] and using cross-sectional study design, that makes this study different as it is conducted at city level and apply case control study design .Therefore the main aim of this study is to identify the determinant factors for inappropriate complementary feeding among 6 to 23 months of age in Dessie city.

## Methods and materials

Study design and setting A community based unmatched case-control study was conducted among mothers/caregivers having children with the age group of 6 to 23 months in Dessie city administration from April 13 to May13/2021G.C. Dessie has 26 kebeles 18 in the urban and 8 rural under a total of 5 sub cities. Children aged 6–23 months of age in the city constituting 4.39% of total population (11,998). In Dessie city, there are 8 public health centers, five private general hospitals and one comprehensive specialized hospital [25].

### Participants, sample size determination and sampling procedures

Mothers/caregivers of children and infants 6–23 months old who were residents of Dessie City for the past 6 months were included in the study. However mothers/caregivers having children at the age of 6 to 23 months having acute or urgent medical need for the child that makes it difficult to interview them and those mothers/caregivers who can’t speak and give organized information were excluded from the study. The assumptions for the sample size calculation were: 80% power, 95% level of confidence, 10% non-response rate [14, 17, 24] and a case: control ratio of 1:1.ANC follow up gives the largest sample with percent of controls exposed 98.2% and percent of cases exposed 89.7% [26], This yield, a total sample size of 321 with 1.5 design effect it yields 482 (241 cases and 241 controls).Simple random sampling technique was employed to select 30% of kebeles on both urban and rural kebeles; which results 6 urban kebeles and 3 rural kebeles. One month prior to data collection preliminary survey was conducted on 3405 children at the age group of 6–23 months in the selected nine kebeles with the standard measurement. Among them 1962 of them were classified under case group (having inappropriate complementary feeding) and the rest 1443 were under the control group (having appropriate complementary feeding) then from the list of identified households, a total of 241 cases and 241 controls were selected by computer generating random numbers (simple random sampling technique).

### Data collection method and tools

The data were collected using a structured interviewer administered questionnaires. The questionnaires were adapted from different literatures of similar studies [16, 17]. The indicator to classify cases and controls was adopted from the indicators developed by WHO [27].

The appropriateness of complementary feeding was assessed by 24 h recall of the indicators. These indicators was timely initiation of complementary feeding by the age of 6–8 months, minimum dietary diversity it is receiving foods from at least five or more food groups out of the eight food groups with in a 24 h time period and the third one is minimum meal frequency it is the proportion of breastfed and non-breastfed children 6–23 months of age, who receive solid, semi-solid, or soft foods for the minimum number of times or more for their respected age [28]. Inappropriate complementary feeding practices: Infants and young children feeding practices that did not satisfy one of the above three criteria of WHO [29].

Household food insecurity was assessed using Household Food Insecurity Access Scale (HFIAS) [30]. The house-hold head was asked a series of nine questions which will address whether the household ran out of food or did not have enough money to buy food in the last one month. If there is a problem they were again asked further on the frequency of the occurrence of the situation as rarely, sometimes or frequently. The questionnaire for assessing household food insecurity is composed of 9 questions each of them has three alternatives as rarely, sometimes and often. That means it was scored out of 27 and finally, households were categorized as food secured when the score was ≤ 1( only for the first question), and food insecure for a score ≥ 2 [30–32].

The questionnaire for assessing knowledge of mothers concerning nutrition and feeding of infants and young children and feeding practice in children was adapted from the Food and Agriculture Organization questionnaires and guiding principles of young child feeding by WHO [33, 34]. The Infant young child feeding (IYCF) knowledge question includes a variety of 14 questions which contains about breastfeeding, complementary feeding and importance of complementary feeding, child feeding during illness, hygiene during feeding and preparing food and ways of making complementary feeds more nutritious. Then it was analyzed by using principal component analysis and divided into three groups poor knowledge, medium knowledge and high knowledge [31].

The questionnaire for wealth index was constructed from composite measure of a household’s cumulative living standard for both the urban and rural areas. It is calculated using easy to calculate data on household’s ownership of selected assets, such as televisions and bicycles; materials used for housing construction; and type of water access and sanitation facilities.

Water, sanitation and hygiene (WASH) index was adopted from FANTA guideline which is composed of 10 questions; 2 questions based on data collector’s observation and the other questions are by asking the mother. Each questions has 2 alternatives which are “yes” or “no” then coded as “1” and “0” respectively then the ten of them were summed and the average was taken as a cut of point to classify them as good hygiene or poor hygiene [35].

### Data quality control

The questionnaire was first prepared in English version then translated into Amharic version by nutrition expert and then again translated back into English version. The data was collected by three diploma nurses and supervised by one health officer. Training was given to the supervisor and data collectors prior to data collection. The tool was pre-tested by administering to 5% of the total sample on population characteristics similar to those of the intended respondents before the actual implementation of the study to see for the accuracy of responses and to estimate the time needed; then appropriate adjustment and corrections was taken accordingly.

### Data processing and analysis

Data were checked, coded and entered into Epi data version 3.1 and then exported to SPSS (Statistical Package for Social science) version 26 for analysis. Descriptive statistics, chi-square test, crude and adjusted odds ratio were used to measure the association between variables and the result was presented with different texts, graphs and tables. Binary logistics regression analysis was carried out to identify the determinant factors for the outcome variables. The decision was made using Odds ratio (OR) and confidence interval (CI) at 95% confidence level. Hosmer-lemenshow test and omnibus test were used to check for model fitness. Cronbach’s alpha coefficient was checked for the composite variable to check for internal consistency between items. Assumptions for logistic regression were checked and fulfilled.

Wealth index (WI) was analyzed by using Principal Component Analysis (PCA) using SPSS version 26. It took steps to determine wealth tertile of respondents. The suitability of the data for factor analysis was assessed by Bartlett’s test of Sphericity and Kaiser-Meyer-Olkin (KMO). The analytic process was based on matrix of correlation between variables. Bartlett’s test of Sphericity was used to test the strength of the relationship between variables. Also Kaiser-Meyer-Olkin (KMO) measured the sampling adequacy to be greater than or equals to 0.5 for each individual variable as well the set of variables. Then factors were extracted by using principal component approach. Factors were rotated by using Varimax Orthogonal approach to present the pattern of loading in a manner that is easier to interpret by identifying variables that have complex structure. After that, new variables were constructed using factor scores for further analysis. Lastly the factor scores were summed and divided into tertile.

Maternal knowledge and perception was also analyzed by using Principal Component Analysis (PCA) using SPSS version 26. Using the same steps with the above (wealth index) then it was classified into three group namely poor, medium and high knowledge.

## Results

### Socio-demographic characteristics

From a total of 482 study participants 460 participants were involved in the study which gives a response rate of 95.4%. Mothers who were in the age category of 25–34 years accounts for 65% in the case group and 62% in the control group. In both groups 97% of the mothers participated were the biological mothers of the respective child. 51% of children in the case group and 62% of children in the control group were from a family size of 4–6. Six point 9% mothers from cases and 4.4% mothers from controls were unable to read and write, on the other side 15.7% from cases and 25.6% from controls were completed college and above. Among all the mothers or caregivers participated in the study, 54% from cases and 46% from controls had no job, 6.5% from cases and 21.7% from controls had a part time job and 39.5% from cases and 32.2% from controls had full time job. Related to fathers’ job, 17% from cases and 28.3% from controls were merchants, 43% from cases and 48.3% from controls were employed in governmental or non-governmental organizations, 21.3% from cases and 6% from controls were daily laborers and 18.7% from cases and 17.4% from controls were not alive. (Table 1).

**Table 1:** Socio-demographic characteristics of mothers having children 6–23 months of age in Dessie city, 2021(n = 460)

Explanatory variable	Category	Cases (N = 230)	Controls (N = 230)
		Frequency (%)	Frequency (%)
Maternal age	18–24	37(16)	44(19)
	25–34	150(65)	144(62)
	35–49	43(19)	42(19)
Religion	Orthodox	152(66)	163(71)
	Muslim	54(23.5)	49(21.3)
	Protestant	24(10.4)	18(7.8)
Residence	Urban	201(87.4)	201(87.4)
	Rural	29(12.6)	29(12.6)
Marital status	Currently married (in union)	176(76.5)	198(86)
	Currently not married*^1^	54(23.5)	32(14)
Child- mother relationship	Biological mother	224(97.4)	224(97.4)
	Care giver	6(2.6)	6(2.6)
Maternal educational status	Can’t read and write	16(6.9)	10(4.4)
	Can read and write	85(37)	55(24)
	Completed primary school	63(27.4)	59(25.6)
	Completed secondary school	30(13)	47(20.4)
	Completed college and above	36(15.7)	59(25.6)
Paternal educational status	Can’t read and write	8(3.5)	5(2.1)
	Can read and write	76(33)	36(15.7)
	Completed primary school	48(20.9)	30(13)
	Completed secondary school	34(14.8)	55(24)
	Completed college and above	64(27.8)	104(45.2)
Mother’s job	Merchant	23(10)	41(17.8)
	Employed*^2^	50(21.7)	71(30.8)
	Daily labourer	33(14.3)	12(5.2)
	House wife	124(54)	106(46.1
Work situation of the mother	Not having job	124(54)	106(46.1)
	Part time job	15(6.5)	50(21.7)
	Full time job	91(39.5)	74(32.2)
Father’s job	Merchant	39(17)	65(28.3)
	Employed*^2^	99(43)	111(48.3)
	Daily labourer	49(21.3)	14(6)
	Not alive	43(18.7)	40(17.4)
Family size	<=3	86(37.4)	69(30)
	4–6	118(51.3)	143(62.2)
	>=7	26(11.3)	18(7.8)
Mothers had known chronic illness	Yes	24(10.4)	15(6.5)
	No	206(89.6)	215(93.5)

*^1^ single, separated, divorced and widowed

*^2^ employed at governmental or private organization

### Maternal knowledge on IYCF and child characteristics

Regarding to maternal knowledge on IYCF, 94.7% of mothers/caregivers knew the need for having good hygiene before preparing and serving food for their child; 85% of them know to increase fluid intake and 77.4% of them knew the need to increase breast feeding frequency during and after child illness. 78.3% of them knew the ways to encourage a child to eat food. Among mothers under the control group; 94% of them knew when to start complementary feeding and among them 72.6% of them knew the importance of initiating complementary feeding; 95% of them knew the need to increase fluid intake and 85% of mothers knew the need to increase breast feeding frequency during and after child illness; Likewise 93.5% mothers knew the need for having good hygiene before preparing and serving food for their child and 85% mothers knew how to encourage their child to have food.

Regarding the consistency of food for the child 67.4% of mothers from the case group and 35% of mothers from the control group picked the picture with thin consistency and the main reason for picking it in both groups was due to their belief that if the food is thin it can be swallowed easily by the child.

Regarding food diversity 50% of the controls knew how to make porridge more nutritious by adding different food groups for the child on the other side only 26% of the cases knew how to make porridge nutritious for their child.

**Fig. 1:**
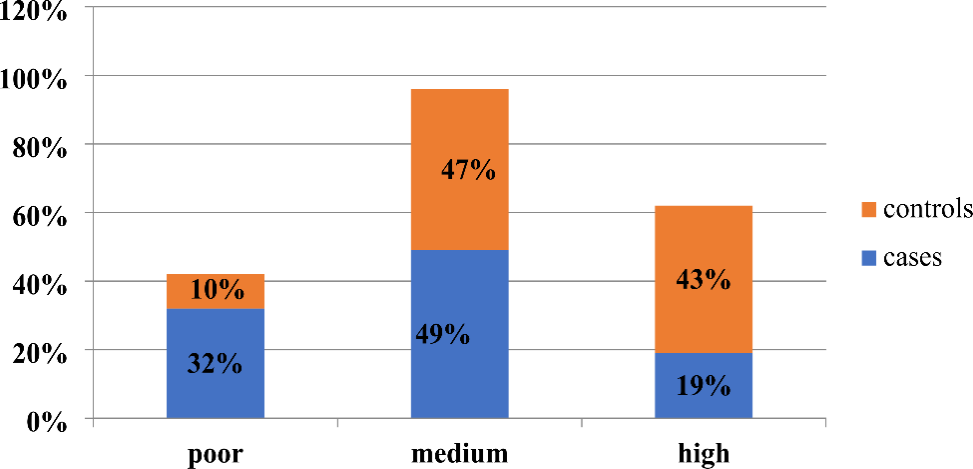
Knowledge on IYCF of mothers having children 6–23 months of age in Dessie City, 2021. Knowledge status of mothers’ on IYCF

The overall knowledge status of the mothers’ on IYCF was classified into three as high, medium and poor knowledge. Both in the cases and controls they had almost similar medium knowledge which is 49% in the cases and 47% in the controls. (Fig. 1).

From the cases 43% and 47% from the control group were males and 57% of study participants from the cases and 53% from the controls were females. Among the cases 40.4% and among the controls 35.2% of them were in the age group of 6–11 months; 33.5% from the cases and 33% from the controls were in the age group of 12–17 months; 26% from the cases and 31.7% from the controls were in the age group of 18–23 months of age. 33% from the cases and 27.3% from the controls were the first child for the family; 20% from the cases and 8.7% from the controls had less than two years age gap from their preceding sibling and 47% from the cases and 64% from the controls had greater than or equal to two years age gap from their preceding sibling. (Table 2).

**Table 2:** Child characteristics of 6–23 months of age children in Dessie city, 2021(n = 460)

Explanatory variable	Category	Cases (N = 230)	Controls (N = 230)
		Frequency (%)	Frequency (%)
Sex	Male	99(43)	108(47)
	Female	131(57)	122(53)
Age	6–11	93(40.4)	81(35.2)
	12–17	77(33.5)	76(33)
	18–23	60(26.1)	73(31.7)
Birth spacing	>= 2 years	108(47)	147(64)
	< 2 years	46(20)	20(8.7)
	First child	76(33)	63(27.3)

### Houshold related factors

Based on wealth status among the cases 46% and from controls 76.5% of them were rich, from the cases 8.6% and from controls 7.4% of them had medium wealth status and 46% of them from the cases and 16.1% from the controls had poor wealth status. 60% of house-holds in the case group and 95% in the control group had secured food status. Most of the children 92.6% in cases and 97% in control groups ate food alone. (Table 3).

**Table 3:** Household characteristics of children aged 6–23 months in Dessie city, 2021(n = 460)

Explanatory variable	Category	Cases (N = 230)	Controls (N = 230)
		Frequency (%)	Frequency (%)
Wealth index	Rich	105(45.7)	176(76.5)
	Medium	20(8.6)	17(7.4)
	Poor	105(45.7)	37(16.1)
Food security	Food secured	139(60.4)	219(95.2)
	Food in secured	91(39.6)	11(4.8)
Presence of television	Yes	139(63.5)	202(87.8)
	No	91(36.5)	28(12.2)
Presence of radio	Yes	171(74)	207(90)
	No	59(26)	23(10)
WASH index	Good WASH	142(61.7)	193(84)
	Poor WASH	88(38.3)	37(16)
Time it takes to fetch water	< 30 min	193(84)	196(85)
	30 min-1 h	22(9.5)	20(9)
	>=1 h	15(6.5)	14(6)
With whom the child feeds	Alone	213(92.6)	223(97)
	With family members	17(7.4)	7(3)

### Obstetrics related factors

One hundred seventy-one (73%) cases and one hundred eighty three(80%) controls of the children were from a mother having parity of three and less and the rest 59(27%) of the children from the case group and 47(20%) from the control group were from mothers having parity of 4 to 6. On the other hand among the cases 36(16%) and among the controls only 7 (3%) were born from unplanned pregnancy. Related to breast feeding status among the cases 57(25%) and among the controls 46(20%) were not breast feeding their children. From the cases 85% and from the controls 87.8% were delivered through non-cesarean mode of delivery (Table 4).

**Table 4:** Obstetrics related factors of mothers’ of children 6–23 months of age in Dessie city, 2021( n = 460)

Explanatory variable	Category	Cases (N = 230)	Controls (N = 230)
		Frequency (%)	Frequency (%)
Parity	<=3	171(73)	183(80)
	4–6	59(27)	47(20)
Type of pregnancy	Planned	194(84)	223(97)
	Unplanned	36(16)	7(3)
Mode of delivery	Vaginal delivery	196(85)	202(87.8)
	Caesarean delivery	34(15)	28(12.2)

### Health-facility related factors

In this study among the cases only 22(9.6%) of them get health education about complementary feeding during their ANC or PNC visit on the other hand 85(37%) of controls get health education about complementary feeding. Among the cases 18(7.8%) of the children are born at home and among the controls only 9(4%) were born at home. Twenty two point 6% of participants from the cases and 68.7% from controls had PNC visit for the index child. (Table 5).

**Table 5:** Health-facility related factors of mothers having children 6–23 months of age in Dessie city, 2021(n = 460)

Explanatory variable	Category	Cases (N = 230)	Controls (N = 230)
		Frequency (%)	Frequency (%)
Time it takes from home to health facility	< 30 min	134(58.3)	157(68.3)
	30 min to 1 h	77(33.5)	53(23)
	>=1 h	19(8.2)	20(8.7)
ANC follow up	Yes	216(94)	221(96)
	No	14(6)	9(4)
Vaccination status	Fully vaccinated	71(30.9)	106(46.1)
	Not fully vaccinated	28(12.1)	7(3)
	Vaccinated for age	131(57)	117(50.9)
Place of delivery	Health facility	212(92.2)	221(96)
	Home	18(7.8)	9(4)
PNC visit	Yes	52(22.6)	158(68.7)
	No	178(77.39)	72(31.3)
HE on CF	Yes	22(9.56)	85(37)
	No	208(90.43)	145(63)

### Dietary diversity of study participants

The 24 h food consumption of all the study participants was assessed and the foods were grouped under the eight food groups. The most consumed food groups among the controls were legumes and nuts which account 85.2%: grains, tubers and roots which account 84%; other fruits and vegetables which account 81% and breast milk which accounts 80%. On the other side the most consumed food groups among the cases were breast milk which account 75.2%; legumes and nuts which account 72.2% and grains, tubers and roots which account 60.4%.

**Fig. 2:**
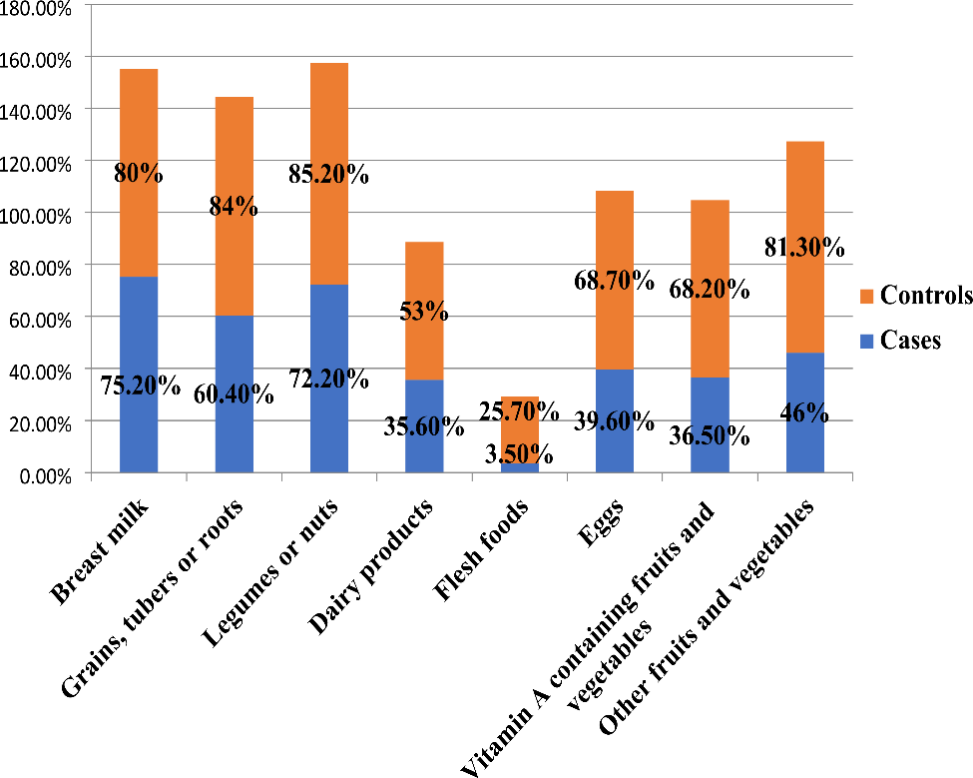
Dietary diversity of children 6–23 months of age among cases and control group in Dessie city, 2021

On the other side the least consumed food groups in both groups is flesh foods which accounts 25.7% in control group and 3.5% in case group. In general all food groups were consumed in much higher percentage in the control groups than the cases. (Fig. 2).

### Determinants of inappropriate complementary feeding among children 6–23 months of age in Dessie city, 2021

On multivariable logistics regression model, those children having a mother/caregiver; with a part time job were found to have 80% reduced odds of having inappropriate complementary feeding when compared with those children having a mother without job [AOR = 0.2 CI(0.084,0.158)].(Table 6).

**Table 6:** Multivariable logistics regression analysis for determinants of inappropriate complementary feeding among children 6–23 months of age in Dessie city, Ethiopia, 2021(n = 460)

Variable	Category	Cases(N)	Controls(N)	COR(95%CI)	AOR(95%CI)	P-value
Work situation of mother	Not having job	124	106	1	1	
	Part time job	15	50	0.256(0.136,0.483)	0.209(0.084,0.158)	0.001*
	Full time job	91	74	1.051(0.703,1.571)	1.708(0.986,2.958)	0.056
Father’s occupation	Merchant	39	65	1	1	
	Employed*^2^	99	111	1.618(0.975,2.685)	2.196(1.172,4.113)	0.014*
	Daily labourer	49	14	9.037(4.386,18.62)	4.571(1.595,13.096)	0.005*
	Not alive	43	40	6.565(3.504,12.29)	5.482(2.488,12.098)	0.000*
Wealth index	Rich	105	176	1	1	
	Medium	20	17	1.972(0.989,3.933)	1.802(0.914,3.556)	0.089
	Poor	105	37	4.757(3.046,7.430)	2.7(1.091,6.681)	0.032*
Food security	Food secured	139	219	1	1	
	Food in secured	91	11	13.034(6.731,25.237)	4.489(1.944,10.365)	0.000*
Place of birth	Health facility	212	221	1	1	
	Home	18	9	2.085(0.916,4.743)	4.334(1.428,13.153)	0.010*
PNC visit	Yes	52	158	1	1	
	No	178	72	7.512(4.955,11.388)	4.062(2.350,7.019)	0.008*
HE on CF	Yes	22	85	1	1	
	No	208	145	5.542(3.313,9.272)	2.540(1.275,5.063)	0.000*
Knowledge on IYCF	High	43	99	1	1	
	Medium	113	108	2.409(1.544,3.758)	1.983(1.104,3.561)	0.000*
	Poor	74	23	7.407(4.11,13.351)	5.942(2.8,12.608)	0.022*

*^2^ employed at governmental or private organization

* P value < 0.05

Those children at the age of 6–23 months having a father with occupation of employed at governmental or non-governmental organization had increased the odds of practicing inappropriate complementary feeding by two times[AOR = 2 CI(1.172,4.113)]; those fathers occupation as a daily laborer increases the odds of having inappropriate complementary feeding by four and half times[AOR = 4.571 CI(1.595,13.096)] and those fathers who are dead had increase the odds of developing inappropriate complementary feeding by five and half times[AOR = 5.482 CI(2.488,12.098)].Those children from house-holds with poor wealth status had 2.7 times higher odds of practicing inappropriate complementary feeding than those children from higher wealth status house hold[AOR = 2.7 CI (1.091,6.681)].

Those children from house-holds with food insecurity status had 4.5 times higher odds of having inappropriate complementary feeding than those children from the house-holds with food security [AOR = 4.489 CI (1.944, 10.365)].

Those children who were born at home had four times higher odds of having inappropriate complementary feeding than those children who were born in the health facility [AOR = 4.334 CI(1.428,13.153).

Those children having a mother with no PNC visit had increased the odds of practicing inappropriate complementary feeding by four times than those children having a mother with PNC visit [AOR = 4.062 CI (2.350, 7.019)].

Those mothers who were not given HE about IYCF during their ANC or PNC visit were found to increase the odds of practicing inappropriate complementary feeding for their children by two and half times [AOR = 2.540 CI(1.275,5.063)].

Those mothers who had medium knowledge on IYCF had twice increased odds of practicing inappropriate complementary feeding than those mothers having high knowledge [AOR = 1.983 CI(1.104,3.561)]. Those mothers who had poor knowledge on IYCF had six times increased odds of developing inappropriate complementary feeding than those mothers having high knowledge on IYCF [AOR = 5.942 CI(2.8,12.608)].

## Discussion

Work situation of the mother was one of the factors affecting appropriateness of complementary feeding. Those mothers working part time had reduced risk of developing inappropriate complementary feeding for their child when compared with mothers who are not working. This result is similar with a research done at Nepal [36]. Which can be related with women empowerment; a working mother is more decision maker in the house than the non-working ones so she can manage the feeding condition of the child. Conversely, in developed countries working mothers initiate complementary feeding before 6 months of age; which makes it inappropriate complementary feeding which is due to their work situation and a belief that considering breast feeding is an old fashioned [37]. In Ethiopia, some researches showed that working mother might wean breast feeding too early to start on her work but non-working mothers may initiate CF at 6 months of age [13].

Father’s job was another factor affecting the appropriateness of complementary feeding. Those children from a family with a father working as employed at governmental and non-governmental; daily laborer and a father who is not alive increase the odds of having inappropriate complementary feeding when compared to those children having a father working as a merchant. This could be related to the fact that merchants in the study area have a relatively high income, which could lead to increased purchasing power and easier access to a variety of foods as they begin complementary food, as opposed to other occupations [38].

Those children from a family with poor wealth index had higher odds of developing inappropriate complementary feeding than from those children from high wealth index. This result is in line with other researches done previously [31, 39, 40]. This may be due to the fact that one of the requirement for achieving dietary diversity is a household’s capacity to pay to buy basic food [41].

Those children from a family having food insecurity had higher odds of developing inappropriate complementary feeding than those children from a family having food security. This finding is supported by other researches done in Kenya [42] and in northwest Ethiopia [32]. This is because in a food in secured house the children might not get diversified and adequate food.

Those children who were born at home had increased odds of developing inappropriate complementary feeding than those children who were born at health facility. This is in line with a research done in Lalibela [23]. This association may be explained by the fact that mothers who gave birth in a health facility were more likely to attend prenatal visits, where they were more likely to develop strong relationships with healthcare professionals who could offer advice and support on appropriate child feeding techniques [16].

Comparing those children having a mother who had no PNC visit had increased odds of developing inappropriate complementary feeding. This result has the same outcome with other researches published previously [40, 43]. This is because mothers who visit postnatal clinics are more likely to get education about the value of providing the child with a healthy diet. This implies that mothers should be encouraged to attend antenatal and postnatal clinics in order to increase their knowledge about infant and young child feeding practices [16].

Knowledge was also one of the factors contributing to have appropriate complementary feeding. This study shows that having poor knowledge on IYCF increases the odds of having inappropriate complementary feeding. This result was in line with other researches [31, 44]. Knowledge had the power to understand and practice the best way by avoiding some irrelevant and misconceptions. If the mother has the knowledge she would practice it without any fear and external influence.

In this study, mothers who had no health education during ANC or PNC visit had increased odds of having inappropriate complementary feeding. This is compatible with other researches [45]. This might be due to the knowledge status of the health professional on IYCF. To increase the knowledge of mothers’ on IYCF health education during ANC and PNC visit is non-replaceable mechanism.

### Limitation of the study

Some important variables were not included in this study like cultural malpractice, male involvement in the house, any support for the family and maternal decision making in the house and food weight was not done.

## Conclusion

From this study we can conclude that work situation of the mother, fathers’ job, post natal visit, wealth index, house hold food security status, mothers’ knowledge on infant and young child feeding, place of delivery and health education on complementary feeding were the determinant factors for inappropriate complementary feeding in children 6–23 months of age in Dessie city.

In order to have appropriate complementary feeding practice among mothers of 6–23 months of age children it is crucial to improve maternal knowledge on infant and young child feeding, promote institutional delivery, intensify utilization of post natal care visit and have to encourage women to have job by themselves to get additional income to support their family.

## Data Availability

The manuscript contains all of the evidence that supports the findings. For further information upon reasonable request, the corresponding author may provide more comprehensive details about the manuscript.

## List of abbreviations

ANC: Antenatal Care
AOR: Adjusted Odds Ratio
CF: Complementary Feeding
CI: Confidence Interval
DRH: Dessie Referral Hospital
EDHS: Ethiopian Demographic and Health Survey
EPI: Expanding program on immunization
HE: Health Education
HFIAS: Household Food Insecurity Access Scale
HH: House Hold
IYCF: Infant Young Child Feeding
MAD: Minimum Acceptable Diet
MDD: Minimum Dietary Diversity
MMF: Minimum Meal Frequency
OR: Odds Ratio
PNC: Post Natal Care
SDGs: Sustainable Development Goals
WHO: World Health Organization
WU: Wollo University
